# Effects of Hypoxia on Proliferation and Differentiation in Belgian Blue and Hanwoo Muscle Satellite Cells for the Development of Cultured Meat

**DOI:** 10.3390/biom12060838

**Published:** 2022-06-16

**Authors:** Sanghun Park, Mick Gagliardi, Geertje Swennen, Arin Dogan, Yuna Kim, Yunhwan Park, Gyutae Park, Sehyuk Oh, Mark Post, Jungseok Choi

**Affiliations:** 1 Department of Animal Science, Chungbuk National University, Cheongju 28644, Korea; pksaho@chungbuk.ac.kr (S.P.); rladbsdk0621@chungbuk.ac.kr (Y.K.); yhp056@chungbuk.ac.kr (Y.P.); pk6247@naver.com (G.P.); osehyuk12@naver.com (S.O.); 2 Mosa Meat B.V., 6229 PM Maastricht, The Netherlands; m.gagliardi@maastrichtuniversity.nl (M.G.); geertje.swennen@maastrichtuniversity.nl (G.S.); arin@mosameat.com (A.D.); 3 Department of Physiology, Maastricht University, 6229 PM Maastricht, The Netherlands

**Keywords:** hypoxia, normoxia, bovine satellite cell, proliferation, differentiation

## Abstract

Among future food problems, the demand for meat is expected to increase rapidly, but the production efficiency of meat, which is a protein source, is very low compared to other foods. To address this problem, research on the development and production of cultured meat as an alternative meat source using muscle stem cells in vitro has recently been undertaken. Many studies have been conducted on myosatellite cells for medical purposes, but studies on alternative meat production are rare. In vitro cell culture mimics the in vivo environment for cell growth. The satellite cell niche is closer to hypoxic (2% O_2_) than normoxic (20% O_2_) conditions. The aim of this study was to investigate the efficient oxygen conditions of myosatellite cell cultures for the production of cultured meat. The bovine satellite cell counts and mRNA (Pax7, Myf5 and HIF1α) levels were higher in hypoxia than normoxia (*p* < 0.05). Through Hoechst-positive nuclei counts, and expression of Pax7, MyoD and myosin protein by immunofluorescence, it was confirmed that muscle cells performed normal proliferation and differentiation. Myoblast fusion was higher under hypoxic conditions (*p* < 0.05), and the myotube diameters were also thicker (*p* < 0.05). In the myotube, the number of cells was high in hypoxia, and the expression of the total protein amounts, differentiation marker mRNA (myogenin, myosin and TOM20), and protein markers (myosin and TOM20) was also high. The study results demonstrated that the proliferation and differentiation of bovine myosatellite cells were promoted more highly under hypoxic conditions than under normoxic conditions. Therefore, hypoxic cultures that promote the proliferation and differentiation of bovine myosatellite cells may be an important factor in the development of cultured meat.

## 1. Introduction

The current world population is about 7.8 billion, and it is expected to reach 9.6 billion by 2050 [1]. Experts predict that, for this reason, the global food demand will increase by more than 70% above the current levels. To prepare for this, environmentally friendly agricultural technologies are being studied very actively around the world [2]. However, due to the uncertainty and unsustainable nature of resources such as water, land, air, and energy caused by climate change, it is predicted that it will be difficult to meet future food demands with the planet’s limited resources [3]. Of the traditional foods, livestock for supplying meat is fed and raised by land covering 30% of the world’s surface [4]. Currently, meat consumption amounts to 360 million tonnes globally, and by 2050, meat demand is expected to increase by 70% [5]. Thus, policies and technologies are being tried in the traditional livestock industry in various countries, but it is necessary to develop a new meat production system that can meet the needs of humans while taking into account environmental issues.

In vitro skeletal muscle tissue is a technology that mimics meat based on the principle of in vivo myogenesis or muscle regeneration after injury [6,7]. Muscle satellite cells, which are muscle precursor cells, are in the stationary state in vivo and exist between the sarcolemma of the muscle and the muscle fibers [8]. Muscle satellite cells play unique roles in maintaining and replenishing the muscle stem cell niche [9,10]. They are primarily responsible for the regeneration of muscle tissue [11,12]. After an injury, muscle regeneration occurs through the activation, proliferation, differentiation, and fusion of muscle satellite cells [13,14,15]. The principle of cultured meat involves the extraction and proliferation of muscle progenitor cells. The proliferated cells in vitro are then differentiated into myotubes and matured into a muscle to become meat. However, to achieve cultured meat production, some challenges need to be solved technically.

Among the important factors in cultured meat production technology are cell source, culture media, scaffolds, animal-derived and synthetic materials, and mimicking the in vivo myogenesis environment (temperature, humidity, dissolved oxygen tension, air composition, etc.) for cell growth [16,17]. In a typical cell culture incubator, the temperature is 37 °C, the humidity is 95% or more, and the CO_2_ concentration is maintained at 5% to maintain the proper pH of the medium. Various in vivo tissues and cells, such as brain, bone, and blood, have their own optimal oxygen level for growth [18]. The partial concentration of oxygen progressively decreases after it enters the lungs and is transported by the blood to reach tissue. Finally, average concentration of physiological oxygen at the tissues ranges between 2 and 9% [19]. The appropriate in vivo oxygen condition for muscle cells was reported to be 1–10% [20,21,22]. Recently, some studies reported the effect of air composition on the proliferation and differentiation of muscle satellite cells. The proliferation of mice myosatellite cells in hypoxic conditions (2% O_2_) showed an increase compared to that of in normoxic conditions [23]. Another study reported that the myogenesis of porcine satellite cells was remarkably improved under hypoxic conditions (5% O_2_) [24]. When human mesenchymal stem cells were cultured for 7 days in 2% oxygen, the cell number increased about 1.5 times more than in normoxic conditions [25]. In addition, Koning et al. (2011) reported that the proliferation of hypoxically cultured human muscle satellite cells (P0–5) was faster than that in the normoxic condition and that P6–15 cells also showed increased proliferation in the transition from normoxia to hypoxia [26]. 

However, the effect of hypoxic conditions on the proliferation and differentiation of bovine muscle satellite cells for cell culture meat development has rarely been studied [27]. Belgian Blue calf is born with twice the number of muscle fibers at birth than a calf without the myostatin gene mutation and has a muscle yield around 20% more on average than a calf without the genetic myostatin mutation [28]. Hanwoo is Korean indigenous Taurine cattle, characterized by marbling, soft texture, juiciness and unique flavor [29]. The aims of this study are to confirm the effect of hypoxic culture using Belgian Blue muscle cells with excellent muscle production yield, and then apply hypoxic culture to the production of cultured meat using Hanwoo myosatellite cells to provide basic data to the cultured meat industry.

## 2. Materials and Methods

### 2.1. Primary Bovine Myosatellite Cell Isolation and Fluorescence-Activated Cell Sorting (FACS)

For primary myosatellite cell cultures, 100 g of muscle tissues were collected from the buttocks of Hanwoo steer at 33 months of age within 30 min after slaughter and transferred to the laboratory on ice. Muscle satellite cells were separated from the muscle with collagenase type II (600 units/mL DMEM) and centrifuged to harvest the cells. The harvested muscle satellite cells were stored in liquid nitrogen by allotting 1 × 10^6^ cells per vial in cell culture freezing medium (Gibco, Waltham, MA, USA) until the experiment. These cells were considered passage 0. Belgian Blue satellite muscle cells were obtained by taking muscle tissues from slaughterhouse in the Netherlands. These cells were also collected through the same process for obtaining satellite cells from Hanwoo muscle tissues.

Prior to FACS, cells were cultured on bovine collagen type I (Cat # C2124, Sigma-Aldrich)-coated flasks and the cells were suspended in FACS buffer (1:100 Bovine Serum Albumin in PBS) and stained with APC anti-human CD29 Antibody (1:10, Cat # 303008, BioLegend), PE-CyTM7 anti-human CD56 (1:10, Cat # 335826, BD), FITC anti-sheep CD31 (1:10, Cat # MCA1097F, Bio-Rad), FITC anti-sheep CD45 (1:10, Cat # MCA2220F, Bio-Rad) for 40 min on ice. After antibody incubation, the cells were washed twice with cold PBS and reconstituted in Ham’s F-10 nutrient mix (Gibco) with 20% fetal bovine serum (FBS) and 1% PSA antibiotics (penicillin-streptomycin-amphotericin B solution, Lonza, Swiss). The positive CD31−, CD45−, CD56+, CD29+ cells were sorted by using FACS Aria II Cell Sorter (BD).

### 2.2. Myosatellite Cell Culture

The growth medium consisted of 20% FBS and 1% PSA antibiotics in Ham’s F-10 nutrient mix. The differentiation medium consisted of 2% FBS and 1% antibiotics (penicillin-streptomycin-amphotericin B solution, Lonza, Swiss) in Dulbecco’s Modified Eagle Medium (Gibco). Hanwoo myosatellite cells were cultured in hypoxic (2% O_2_, 5% CO_2_, 93% N_2_) and normoxic (20% O_2_, 5% CO_2_, 75% N_2_) conditions using growth medium (GM) for proliferation, and differentiation medium (DM) for differentiation. The atmospheric composition of the incubators (Hanwoo myosatellite cells were cultured in CelCuture^®^ CO2 Incubators, ESCO, and Belgian Blue myosatellite cells were cultured in Great CO2 Incubator for Cultured Cells, Sanyo) were set by injecting CO_2_ and N_2_ gas. The satellite cells were seeded at 1800 cells/cm^2^ and incubated in a humidified incubator at 37 ℃. The satellite cells were cultured in GM until confluent on the 96-well plate or T25 flask and then differentiated into muscle myotubes in DM. Cells cultured with GM only were seeded on pre-coated bovine collagen type I flasks, and cells replaced with DM from GM were seeded into pre-coated Matrigel flasks (Cat # 354234, Matrigel^®^ Basement Membrane Matrix, Corning^®^, Herndon, VA, USA).

### 2.3. Cell Proliferation Assay

The cells were cultured under normoxic or hypoxic conditions. Cell proliferation assay was evaluated using CellTiter 96^®^ AQueous One Solution Cell Proliferation Assay (Promega, Madison, WI, USA). The assay consists of colorimetric [3-(4,5-dimethylthiazol-2-yl)-5-(3-carboxymethoxyphenyl)-2-(4-sulfophenyl)-2H-tetrazolium (MTS)], which reduces to Formazan by dehydrogenase enzyme activity of viable cells. Formazan is soluble in cell culture medium. MTS (final concentration, 0.33 mg/mL MTS) was added to the cells of 96-well plates, incubated at 37 °C for 2 h, and the absorbance was measured at 490 nm.

### 2.4. Cell and Cell Nuclei Counting

The Hanwoo myosatellite cells were detached with trypsin, and the number of cells stained with trypan blue was counted using a cell counter (Countess^®^ cell FL automated cell counter, Invitrogen, USA) after trypsin neutralization solution (2% FBS in PBS) treatment to neutralize trypsin.

The Belgian Blue cells cultured in BD Falcon 96-well HTS Imaging microplates were imaged using a High-Content Analyzer (BD Pathway 855) with a 4× objective. In total, 16 images were taken for each condition, and individual cell segmentation and analysis were performed using the BD Attovision software (BD Biosciences, San Jose, CA, USA, version 1.6). Hoechst-positive cells were segmented and counted. The numerical data were further analyzed with Kaluza software (version 1.3; Beckman Coulter, Brea, CA, USA).

### 2.5. Immunofluorescence Staining

The cells were fixed in 2% paraformaldehyde (in PBS) at 37 °C for 45 min. The fixed cells were permeabilized in 0.1% Triton-X (in PBS) at room temperature for 20 min and blocked with 2% bovine serum albumin (BSA, Roche, New Zealand) at room temperature for 30 min. The cells were treated with diluted Pax7 (1:100, Cat # PA5-68506, Invitrogen, Waltham, MA, USA), MyoD (1:200, Cat # bs-2442R, Bioss, USA), and myosin (1:100, Cat # M4276, Sigma, St. Louis, MO, USA) antibodies at 4 °C overnight after washing the fixed cells with PBS. The cells were then incubated with secondary goat anti-rabbit IgG cross-adsorbed antibody (1:1000, Cat # A21121, Invitrogen) and secondary goat anti-mouse IgG1 cross-adsorbed antibody (1:2000, Cat # A11008, Invitrogen) at room temperature for 30 min. After secondary antibody staining, Hoechst 33,342 (Cat # H3570, Invitrogen) was diluted 2000 times in PBS and used to stain the nuclei for 2 min at room temperature.

### 2.6. Fusion Index

Hanwoo myosatellite cells were cultured in GM for 5 days and then replaced with DM for 3 days. Cell differentiation was investigated on the first and third days, with the day the medium was replaced with DM designated as day 0. On days 1 and 3 of differentiation, the percentage of nuclei in the myotube to the total number of nuclei produced was calculated. The nuclei were confirmed by Hoechst staining (1:2000 dilution in PBS). For each fusion index, five values per repetition were expressed as the mean and standard deviation. This experiment was repeated three times [30,31]. 

### 2.7. Myotube Diameter

On days 1 and 3 of Hanwoo myosatellite cell differentiation, myotubes were confirmed by the immunofluorescence staining of myosin. The diameter of the myotubes was measured using the ImageJ program (NIH, Bethesda, MD, USA). The myotube diameters that were differentiated under hypoxic or normoxic conditions were expressed as the mean and standard deviation of 10 myotubes per repetition. The experiment was performed 3 times [32].

### 2.8. Total Protein and Western Blotting

Total proteins were either performed from total Belgian Blue myosatellite cell lysates obtained by lysing cells directly with RIPA buffer complemented with PMSF, protease inhibitor cocktail and sodium orthovanadate (Cat: sc-24948, Santa Cruz). Proteins concentration was determined using a BCA protein assay kit (Thermo).

For Western blot analysis, Hanwoo cells were collected in sodium dodecyl sulfate (SDS) sample buffer and heated for 5 min at 95 °C. Proteins were separated by SDS-polyacrylamide gel electrophoresis and electrically transferred onto polyvinylidene fluoride (PVDF) membranes. The membranes were blocked in EveryBlot Blocking buffer (Cat: BR12010020, Bio-Rad, Hercules, CA, USA) for 1 h and then incubated overnight at 4 °C with primary antibodies. The primary antibodies used were monoclonal anti-β-actin antibody produced in mice (1:3000, Cat: A2228, Sigma-Aldrich), myosin 4 monoclonal antibody (1:1000, Cat: 14-6503-82, Invitrogen), Tom20 Antibody (F-10) (1:1000, Cat: sc-17764, Santa Cruz). After washing three times with TBST (each for 10 min), the membranes were incubated for 1 h at 37 °C with affinity-purified goat anti-mouse IgG (H+L) horseradish peroxidase (HRP)-conjugated secondary antibody (1:3000, Cat: BR1706516, Bio-Rad). Finally, the membranes were exposed to Clarity Western ECL Substrate (Bio-Rad). Blots were visualized using a CCD camera and UVISoft software (UVITEC Cambridge).

### 2.9. Real-Time Reverse Transcription (RT)-Quantitative PCR

The cultured cells were collected, and total RNA was extracted using the Total RNA Extraction Kit (Cat # 17221, iNtRON Biotechnology, Gyeonggi, Seongnam, Korea) according to the manufacturer’s instructions. cDNA was obtained using the High Capacity cDNA Reverse Transcription Kit (Cat #4368814, Thermo Fisher). Real-time quantitative PCR was performed using Fast qPCR 2x SYBR Green Master Mix (Cat # EBT-1821). Amplification was conducted as follows: 50 °C for 2 min and 95 °C for 10 min, followed by 40 cycles of 95 °C for 15 s, 52–55 °C for 1 min. The target genes were GAPDH, Pax7, Myf5, MyoD, HIF1α, myosin, and TOM20. GAPDH was used as an internal control for RT-PCR. The primers used to amplify each gene were shown in Table 1. The mRNA quantities were analyzed using the 2^−ΔΔCT^ method [33]. 

### 2.10. Statistical Analysis

All statistical analyses including the Student’s *t*-test were carried out using SAS Statistical Package 9.4 (SAS, Cary, NC, USA, 2003). *p*-values of <0.05 indicated significant differences.

## 3. Results

### 3.1. High Proliferative Capacity in Long-Term Cultures of Belgian Blue Muscle Satellite Cells Cultured under Hypoxic Conditions

**Figure 1:**
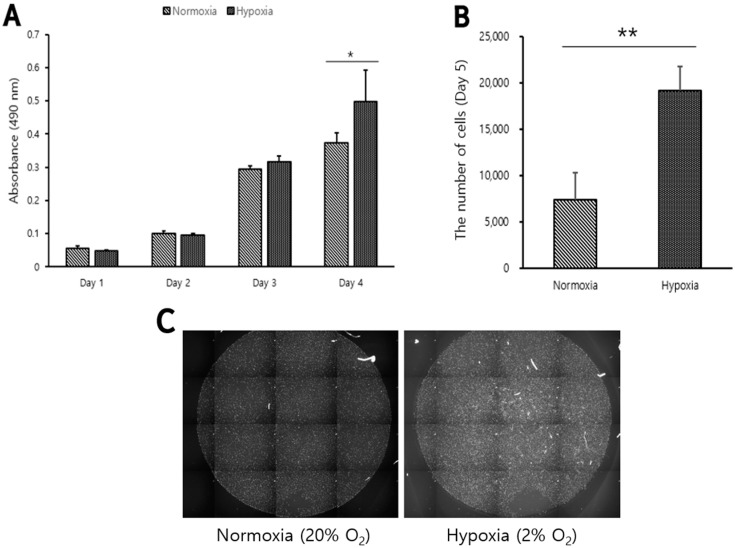
Proliferation of Belgian Blue myosatellite cells under normoxia (20% O_2_) or hypoxia (2% O_2_). (**A**) is quantitative assessment of cell proliferation of Belgian Blue myoblasts cultured on 1, 2, 3 and 4 days under hypoxia (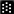) or normoxia (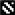) in a 96-well plate. (**B**) is the number of Belgian Blue myoblasts cultured for 5 days in a multiplate under hypoxia or normoxia. (**C**) is the representative image showing Belgian Blue myoblasts cultured for 5 days and cell nuclei stained with Hoechst. The Belgian Blue myosatellite cells were proliferated by culturing in GM. Experiments were performed in triplicate and repeated three times with similar results and values are mean ± standard deviation. Asterisk means significant difference (* *p* < 0.05, ** *p* < 0.01).

To investigate the effect of oxygen conditions on the proliferation of Belgian Blue muscle satellite cells, cell proliferation was confirmed by the MTS assay at normoxia (20% O_2_) and hypoxia (2% O_2_). The cell proliferation under the two conditions was not significantly different on the first, second, and third days of culture, but was significantly higher in hypoxia on the fourth day (*p* < 0.05, Figure 1A). The number of cell nuclei in 5 days of cell culture was confirmed under the two conditions through High Content Analyser. The number of cell nuclei stained with Hoechst measured from Figure 1C was significantly higher in hypoxia (*p* < 0.05, Figure 1B). Belgian Blue myosatellite cells showed a significant difference after the fourth day of culture in hypoxia. It is judged that, the longer the culture period of Belgian Blue myosatellite cells, the greater the difference in cell count.

### 3.2. Belgian Blue Muscle Cells Cultured under Hypoxic Conditions Have more Myotube Formation in Differentiation Phases

**Figure 2:**
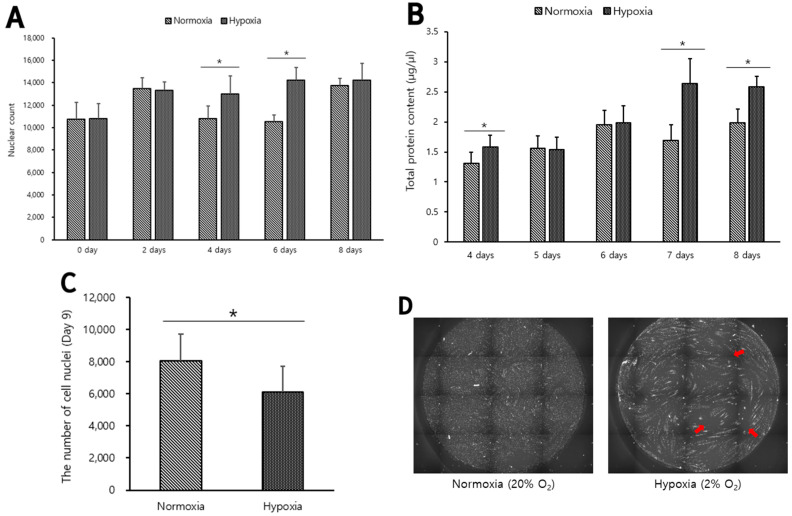
GM in confluent Belgian Blue myosatellite cell culture were replaced with DM and cultured in normoxic or hypoxic conditions during the differentiation phase. In the differentiation phase, Belgian Blue muscle cells were cultured with DM for 0, 2, 4, 6 and 8 days under hypoxia (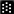) or normoxia (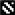) (**A**). From day 4 to day 8, the total protein content of differentiated Belgian Blue was measured (**B**). Belgian Blue muscle cells differentiated for 9 days under hypoxia (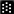) or normoxia (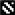) and stained with Hoechst were counted by HCA (**C**). (**D**) is the representative images of Belgian Blue muscle cells stained with Hoechst that produced (**C**) results. Each sample repeated 3 times with triplicate and values are mean ± standard deviation. Asterisk means significant difference (* *p* < 0.05).

Belgian Blue muscle cells were cultured to investigate the effect of oxygen conditions on the differentiation phase of Belgian Blue muscle cells. Confluent cells cultured in normoxia (20% O_2_) were replaced with DM and cultured under normoxic (20% O_2_) or hypoxic (2% O_2_) conditions during the differentiation phase. In the fourth and sixth days of the differentiation phase, the number of muscle cell nuclei was significantly higher in hypoxic condition, and in the eighth day of the differentiation phase, there was no significant difference in the number of muscle cell nuclei (*p* < 0.05, Figure 2A). The total protein content in Belgian Blue muscle cells was measured from the fourth day of differentiation phase, which had a significant difference in the results of Figure 2A. The total protein content was significantly higher in hypoxic conditions of the fourth, seventh, and eighth days of the differentiation phase (*p* < 0.05, Figure 2B). In the ninth day of the differentiation phase, the number of Hoechst-positive cells was significantly higher in normoxia than in hypoxia (*p* < 0.05, Figure 2C). The nuclei of the myotubes were not stained, so the nuclei of the myotubes indicated by red arrows in Figure 2D were not counted. The reason why the total protein content of muscle cells in hypoxia is high on the eighth day of differentiation phase in Figure 2B, but there is no difference in the number of muscle cell nuclei in Figure 2A, is that there are many unstained cell nuclei in the myotubes. Since the protein density of myotubes is higher than that of myoblasts, these results are considered that more myotubes are formed under hypoxic condition than normoxic condition. We confirmed the possibility that the cultured meat production efficiency could be increased through hypoxia culture of Belgians Blue muscle cells.

### 3.3. High Proliferative Capacity in Long-Term Cultures of Hanwoo Muscle Satellite Cells Cultured under Hypoxic Conditions

**Figure 3:**
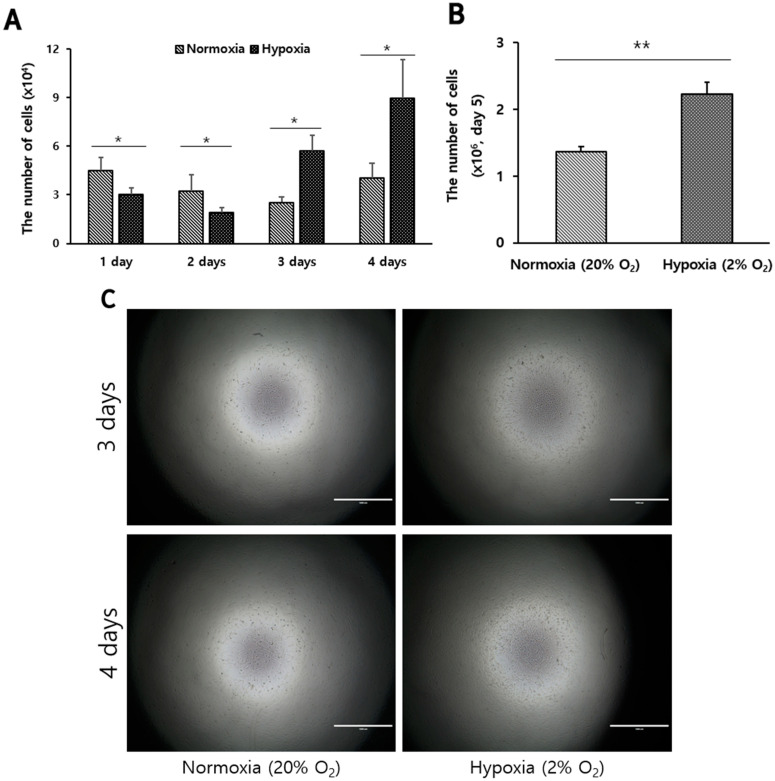
Proliferation of Hanwoo myosatellite cells under hypoxia or normoxia. (**A**) is the number of Hanwoo myoblasts cultured on 1, 2, 3 and 4 days under hypoxia (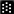) or normoxia (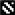) in a 96-well plate. (**B**) is the number of Hanwoo myoblasts cultured for 5 days in T25 flask under hypoxia or normoxia. (**C**) is representative images of the Hanwoo myosatellite cells cultured for 3 and 4 days under hypoxia or normoxia of (**A**) results. The Hanwoo myosatellite cells were proliferated by culturing them in growth medium. Each sample was repeated 3 times with triplicate and values are mean ± standard deviation. Asterisk means significant difference (* *p* < 0.05, ** *p* < 0.01). Scale bar: 1000 μm.

To determine the effect of oxygen conditions on the growth of Hanwoo myosatellite cells, cell proliferation was investigated at a normoxic condition (20% O_2_) and a hypoxic condition (2% O_2_). Under different oxygen concentrations, 5000 cells were seeded in 96-well plates and cultured in GM for 4 days. The cell numbers were higher under normoxic conditions than under hypoxic conditions on the first and second days of culture. However, from the third day of culture, cell growth was increased under hypoxic conditions and was higher under hypoxic conditions than normoxic conditions on days 3 and 4 (*p* < 0.05, Figure 3A,C). Subsequently, to determine the effect of increasing the culture capacity, myosatellite cells were seeded in T25 flasks at 1800 cells/cm^2^ and cultured in GM for 5 days in hypoxia or normoxia. The number of cells was more than 1.5 times higher in hypoxia than normoxia (*p* < 0.01, Figure 3B). As the cell culture period progressed, the cell proliferation increased more under hypoxic conditions than under normoxic conditions. Hanwoo myosatellite cells showed a significant difference after the third day of culture in hypoxia.

### 3.4. Comparison of Relative Proliferation mRNA Levels and Cell Proliferation Proteins Immunofluorescence Staining in Hanwoo Myosatellite Cells

**Figure 4:**
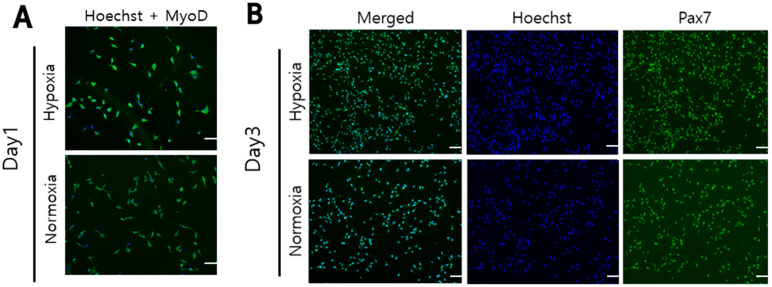

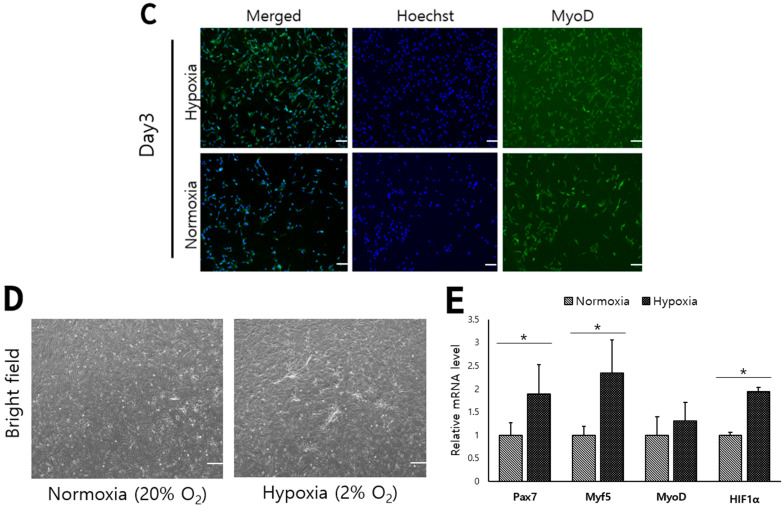
The cell nuclei stained with Hoechst (Hoechst 33,342 nucleic acid stain) were blue fluorescence and Pax7 and MyoD proteins were stained with green fluorescence. (**A**): The nuclei and MyoD protein of Hanwoo myosatellite cells cultured for 1 day in hypoxia (2% O_2_) and normoxia (20% O_2_) were stained. (**B**,**C**): The nuclei, Pax7 and MyoD protein of Hanwoo myosatellite cells cultured for 3 days in hypoxia (2% O_2_) and normoxia (20% O_2_) were stained. Experiments were performed in triplicate and repeated three times (* *p* < 0.05). Hanwoo myosatellite cells were seeded in T25 flasks at 1800 cells/cm^2^ and cultured in GM for 6 days in normoxia (20% O_2_) or hypoxia (2% O_2_) (**D**). (**E**): Relative Pax7, Myf5, MyoD and HIF1α mRNA levels were compared in Hanwoo myosatellite cells from (**D**). GAPDH was used as an internal control for RT-PCR. scale bar: 100 μm.

Immunofluorescence staining was carried out to analyze the distribution of cell nuclei, and expression of Pax7 and MyoD protein in Hanwoo myosatellite cells cultured in both oxygen concentrations. Hanwoo myosatellite cells were cultured for 1 day and 3 days under hypoxic (2% O_2_) and normoxic (20% O_2_) conditions. On the first day of culture, the Hoechst-positive nucleus and MyoD expression areas of Hanwoo myosatellite cells appear to be similar in hypoxia and normoxia (Figure 4A). On the third day of culture, it was confirmed that Hanwoo myosatellite cells were proliferating through the expression of nuclei, Pax7, and MyoD (Figure 4B,C). All cells of Hoechst-positive nuclei contain Pax7 and MyoD protein expression. The proliferation levels of Hanwoo myosatellite cells were measured through the relative levels of Pax7, Myf5, MyoD and HIF1α mRNA. Pax7 is a self-renewing protein of myosatellite cell and Myf5 is a myogenesis regulatory protein. MyoD is involved in the activation and differentiation of muscle cells. It is also important to consider that hypoxia-inducible factor-1α (HIF-1α) signaling pathway is regarded as a key signaling pathway involved in hypoxia-induced responses. Many studies suggest that HIF-1α upregulates the downstream target genes involved in cell proliferation and differentiation. HIF-1α also induces transcription of genes involved in cell proliferation and survival [34]. When cultured in both conditions for 6 days, the Hanwoo myosatellite cells became confluent (Figure 4D). In hypoxia, the Pax7 and HIF1α mRNA level was about twice as high, and the Myf5 mRNA level was more than twice as high as in normoxia (*p* < 0.05, Figure 4E). There is no significant difference in the mRNA level of MyoD. 

### 3.5. Comparison of Muscle Cell Differentiation Marker mRNA and Muscle Differentiation Protein in Hypoxic Conditions

**Figure 5:**
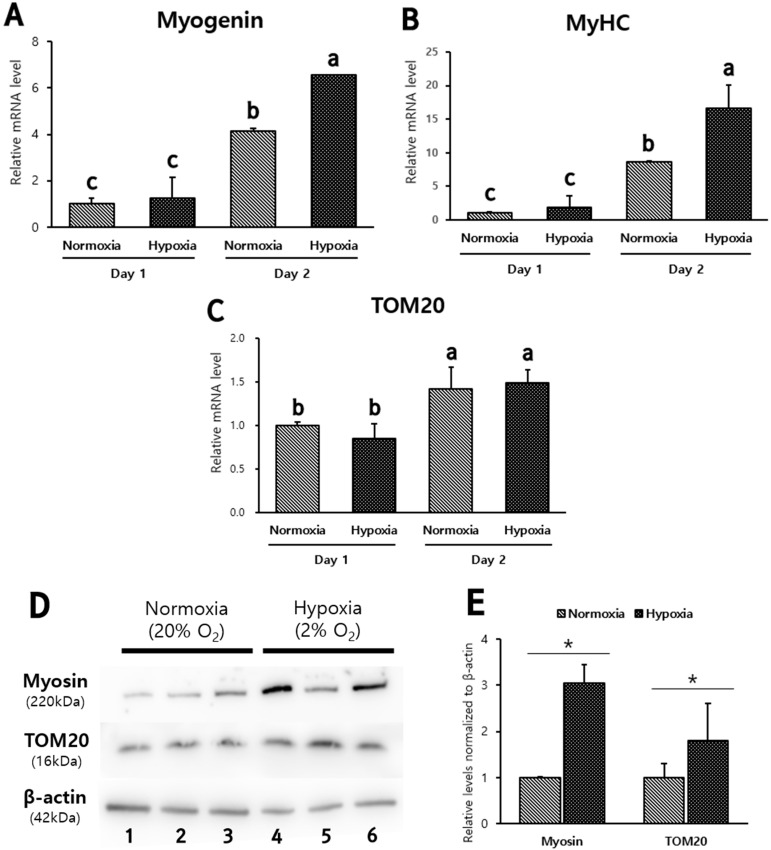
The confluent cells were cultured under hypoxia or normoxia for 1 and 2 days. Myogenin (**A**), MyHC (**B**) and TOM20 (**C**) mRNA levels were relatively analyzed. GAPDH was used as an internal control for RT-PCR. Experiments were performed in triplicate and repeated three times. Mean ± standard deviation with differing letters differ significantly (*p* < 0.05). The confluent Hanwoo muscle cells were cultured for 2 days in DM. (**D**) is representative images of Western blotting against Myosin, TOM20 and β-actin from lysates of differentiated Hanwoo muscle in hypoxia (2% O_2_) (Lane 1, 2, 3) and in normoxia (20% O_2_) (Lane 4, 5, 6). (**E**) is relative levels of myosin and TOM20 normalized to β-actin from (**D**). Asterisk means significant difference (* *p* < 0.05) (*n* = 3).

A quantitative comparison of myocyte differentiation was conducted in hypoxic and normal conditions by analyzing Hanwoo muscle cell differentiation marker mRNA levels. On the first and second days of differentiation culture, the myogenin, myosin heavy chain (MyHC) and translocase of the outer membrane (TOM20) mRNA were analyzed. Myogenin and MyHC protein are both markers of myocyte differentiation. TOM20 protein is one of the receptor systems of the TOM complex in the outer mitochondrial membrane. The myogenin and MyHC mRNA levels of Hanwoo myocytes differentiated for 2 days were significantly higher under hypoxia than normoxia (*p* < 0.05, Figure 5A,B). The myogenin and MyHC mRNA levels of Hanwoo muscle cells differentiated in hypoxia for 2 days were 1.5 times higher than in normoxia (*p* < 0.05, Figure 5A,B). There was no difference in the TOM20 mRNA levels of differentiated Hanwoo muscle cells for 1 and 2 days (Figure 5C). The relative intensity of the protein band determines the amount of myotube formation in Hanwoo muscle cells cultured under hypoxia and normoxia (Figure 5D). Myosin protein, a marker of myocyte differentiation, and TOM20 protein, a receptor subunit of outer mitochondrial membrane, were relatively higher in hypoxic conditions than in normoxic conditions (*p* < 0.05, Figure 5E).

### 3.6. Differentiation of Hanwoo Myoblasts into Myotubes and Their Characteristics in Hypoxia and Normoxia

**Figure 6:**
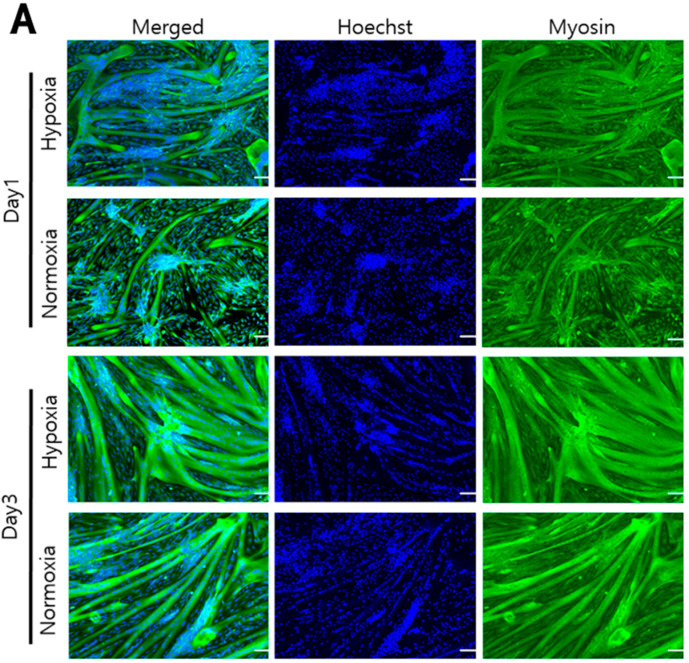

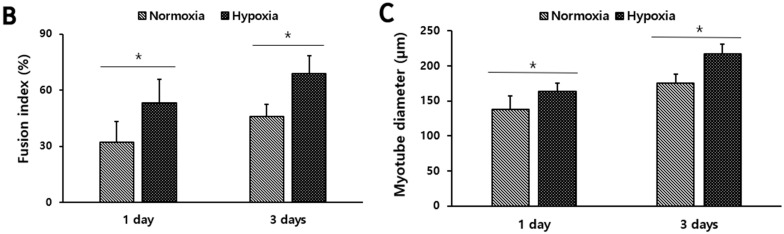
Differentiation of Hanwoo myoblasts were cultured for 1 and 3 days under hypoxia (2% O_2_) or normoxia (20% O_2_). (**A**): The Hanwoo myoblasts were cultured in DM under hypoxia or normoxia for 1 and 3 days. The cell nuclei stained with Hoechst (Hoechst 33,342 nucleic acid stain) were blue fluorescence and myosin proteins in myotubes were stained with green fluorescence. (**B**): On days 1 and 3 of differentiation culture in DM under hypoxia (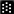) or normoxia (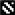), fusion index was calculated as the percentage of nuclei within myotube area to the total number of nuclei produced from (**A**). (**C**): Diameter (nm) of Hanwoo myotubes was differentiated under hypoxia (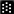) or normoxia (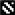) for 1 and 3 days. The diameters of myotube which were differentiated under hypoxia or normoxia were measured using Image J software. Asterisk means significant difference (* *p* < 0.05). scale bar: 100 μm.

To investigate how both oxygen concentrations affected the differentiation of Hanwoo myosatellite cells, the confluent cells were cultured under hypoxia or normoxia in DM for 1 and 3 days. After cell culture in DM for 1 and 3 days, myotube formation was investigated by immunofluorescence of myosin protein. In both cell culture conditions, differentiation resulted in the fusion of myosatellite cells to form multinuclear myotubes. Myosin protein expression was higher in hypoxia compared to normoxia and promoted more myotube formation under hypoxic conditions (Figure 6A). To determine the degree of muscularization in both oxygen environments, the fusion index and the myotube diameter were investigated in myocytes cultured in DM for 1 and 3 days. The fusion index was higher in hypoxia (50%) than in normoxia (30%) on day 1 and higher in hypoxia (70%) than in normoxia (45%) on day 3 of cell culture in DM (*p* < 0.05, Figure 6B). The myotube diameters increased in both oxygen conditions, but their diameters in hypoxia were greater than those in normoxia on both days 1 and 3 of cell culture in DM (*p* < 0.05, Figure 6C). The differentiation of Hanwoo myocytes into myotubes was stimulated more under hypoxic conditions than under normoxic conditions.

## 4. Discussion

The proliferative capacity of Belgian Blue and Hanwoo myosatellite cells were increased under hypoxic conditions compared to normoxic conditions. In particular, the increase in muscle cell proliferation under hypoxic condition was noticeable after 3–4 days of culture. It seems that some culture period is needed to confirm the difference in cell proliferation under both oxygen concentrations. In the early stages of cell division, the satellite cell transformation is limited, and the proliferation and expansion of satellite cells in vivo peaks at 3–4 days [35,36]. During the proliferation peak period of the satellite cells, it seems that the proliferation of cells cultured under both oxygen concentrations was significantly different. These results indicate the significant role that hypoxia plays as a key component in the proliferation of myosatellite cells. In a previous study by Parrinello et al., mouse embryonic fibroblasts senesce was the result of oxidative stress, and the study explained their lack of proliferative ability in long-term culture [37]. There is a possibility of a lack of proliferative power in the long-term culture of Belgian Blue and Hanwoo myosatellite cells. Therefore, an appropriate culture period is required to obtain the advantage of hypoxia culture. The expression of Pax7 was higher in hypoxia when the satellite cells were cultured for 3 days in hypoxia or normoxia. These results indicated that the satellite cells were proliferated into myoblasts and activated more in hypoxia than in normoxia. While quiescent myosatellite cells express the Pax7 gene, the satellite cells first proliferate and express both Pax7 and MyoD. And then, the satellite cells downregulate Pax7 expression but maintain MyoD expression throughout the myogenic differentiation process [38]. Myf5 mRNA is expressed in most quiescent satellite cells, but translation is inhibited [39], and Myf5 and MyoD play important and overlapping roles in the determination, differentiation, and regeneration of myocytes [40]. In both oxygen concentrations, all Hanwoo muscle cells showed that Hoechst-positive nuclei, MyoD, and Pax7 protein expression were merged, and it was judged that cell proliferation and functions of Pax7 and MyoD proteins were performed normally. Through the expression of Pax7 and MyoD proteins on the first and third days of culture, it was confirmed that cell proliferation continued over time. On sixth day of Hanwoo cell culture, Pax7 and Myf5 mRNA levels were high in hypoxia. Therefore, it can be determined that the number of cells is quantitatively high and the proliferative and self-renewal levels are excellent in hypoxia. It is known that the HIF-α transcription factor complex is located in the hypoxic reaction element (HRE) of the target gene to regulate transcription upward. In hypoxia, Hanwoo muscle cells showed higher HIF-α mRNA levels, so there is a possibility of transcription upregulation, and further studies are needed. If Hanwoo myosatellite cells are used for cultured meat production, the production will increase when cultured for more than 3 days to obtain high cell proliferation effect in hypoxia.

Myosatellite cells proliferate if fibroblast growth factor or another growth factor is present in the medium surrounding the cells. Thus, serum deprivation is often used to differentiate myoblasts into myotubes. The satellite cell differentiation proceeds to start cell cycle exit and to enter into myogenesis. Myogenesis involves the alignment of mononucleated myoblasts with one another and aggregation by the parallel apposition of their membranes [41]. Then, membrane fusion occurs, and the cells fuse to form multinucleated tubes [42]. In this experiment, to differentiate myoblasts after proliferating bovine myosatellite cells, a serum-deprived DM, which contained 2% fetal bovine serum, was used in hypoxia or normoxia. The differentiation of Belgian Blue muscle cells is effective when cultured under hypoxia for more than 4 days. Based on the number of cells and the amount of protein, Belgian Blue muscle cells were judged to have an advantage in myotube formation in hypoxia. The differentiation capacity advantage of hypoxia was confirmed in more detail using Hanwoo muscle cells. The relative level of myogenin mRNA, which determine muscle differentiation, and MyHC mRNA were higher in hypoxia. Because there was no significant difference in the TOM20 mRNA level of Hanwoo muscle cells cultured in both oxygen concentrations, TOM20 protein was additionally confirmed. On the second day of differentiation culture of Hanwoo muscle cells, myosin and TOM20 protein were higher in hypoxia. Myotubes and mitochondria of hypoxic cultured Hanwoo muscle cells were quantitatively higher than those of normoxic culture. The myotube formation of Hanwoo muscle cells is effective from the second day of hypoxia differentiation culture. In both oxygen concentrations, it is judged that Hanwoo muscle cells performed cell differentiation and myotube formation normally through Hoechst positive nuclei and myosin protein expression. The increase in myosin protein expression, fusion index, and myotube diameter on the first and third days of differentiation culture shows that muscle differentiation and myotube maturation are continuously progressing over time. When producing cultured meat using Hanwoo muscle cells in hypoxia, the production will increase when cultured for more than 2 days to obtain a high differentiation effect. 

In conclusion, this study proved evidence that more proliferation and differentiation of Belgian Blue and Hanwoo myosatellite cells was promoted in hypoxia than in normoxia. In particular, when cultured meat is produced using Hanwoo muscle cells, culturing in hypoxia can increase cell proliferation by 1.5–2 times for 5–6 days and myotube formation in differentiated culture is 1.5–3 for 2–3 days compared to normoxia. Furthermore, if the production yield of Hanwoo muscle cells is systematized according to oxygen concentration, oxygen concentration will be a factor that controls the production rate of cultured meat. Therefore, these results can be applied to the production of cultured meat as an alternative meat.

## Figures and Tables

**Table 1:** Sequences of the primers used in the RT-qPCR study.

Primer	Sequence (5′-3′)
**Pax7**	F: CTCCCTCTGAAGCGTAAGCA R: GGGTAGTGGGTCCTCTCGAA
**Myf5**	F: AGGATCCAGCCTCTCTCTCC R: TTGCTTTGGGGTTTTTGGTA
**MyoD**	F: GCAACAGCGGACGACTTCTA R: AGGGAAGTGCGAGTGTTCCT
**HIF-1ɑ**	F: AGGACAAGTCACAACAGGAC R: AAAATCAAGTCGTGCTGAAT
**Myogenin**	F: AGAAGGTGAATGAAGCCTTCGA R: GCAGGCGCTCTATGTACTGGAT
**Myosin**	F: CGACAAGATCGAGGACATGG R: AGATGGAGAAGATGTGGGGC
**TOM20**	F: GAATATGAGAAGGGTGTGGA R: AATTGTTGGGAGCTTAGTCA
**GAPDH**	F: CACCACCATGGAGAAGGCCG R: GAACACGGAAGGCCATGCCA

## Data Availability

Not applicable.
